# Geometrical Measures Obtained from Pretreatment Postcontrast T1 Weighted MRIs Predict Survival Benefits from Bevacizumab in Glioblastoma Patients

**DOI:** 10.1371/journal.pone.0161484

**Published:** 2016-08-24

**Authors:** David Molina, Julián Pérez-Beteta, Alicia Martínez-González, Juan M. Sepúlveda, Sergi Peralta, Miguel J. Gil-Gil, Gaspar Reynes, Ana Herrero, Ramón De Las Peñas, Raquel Luque, Jaume Capellades, Carmen Balaña, Víctor M. Pérez-García

**Affiliations:** 1 Laboratory of Mathematical Oncology (MôLAB), Instituto de Matemática Aplicada a la Ciencia y la Ingeniería, Edificio Politécnico, Avda. Camilo José Cela 3, Universidad de Castilla-La Mancha, 13071 Ciudad Real, Spain; 2 Medical Oncology Service, Hospital Universitario, 12 de Octubre, Madrid, Spain; 3 Medical Oncology Service, Hospital Sant Joan de Reus, Reus, Spain; 4 Medical Oncology Service, Institut Catalá d’Oncologia IDIBELL, Hospitalet de Llobregat, Barcelona, Spain; 5 Medical Oncology Service, Hospital Universitario La Fe, Valencia, Spain; 6 Medical Oncology Service, Hospital Miguel Servet, Zaragoza, Spain; 7 Medical Oncology Service, Hospital Provincial de Castellón, Castellón, Spain; 8 Medical Oncology Service, Hospital Universitario Virgen de las Nieves, Granada, Spain; 9 Neuroradiology Section. Radiology Service. Hospital del Mar, Barcelona, Spain; 10 Medical Oncology Service, Institut Català d’Oncologia, IGTP, Hospital Universitari Germans Trias i Pujol, Badalona, Spain; George Washington University, UNITED STATES

## Abstract

**Background:**

Antiangiogenic therapies for glioblastoma (GBM) such as bevacizumab (BVZ), have been unable to extend survival in large patient cohorts. However, a subset of patients having angiogenesis-dependent tumors might benefit from these therapies. Currently, there are no biomarkers allowing to discriminate responders from non-responders before the start of the therapy.

**Methods:**

40 patients from the randomized GENOM009 study complied the inclusion criteria (quality of images, clinical data available). Of those, 23 patients received first line temozolomide (TMZ) for eight weeks and then concomitant radiotherapy and TMZ. 17 patients received BVZ+TMZ for seven weeks and then added radiotherapy to the treatment. Clinical variables were collected, tumors segmented and several geometrical measures computed including: Contrast enhancing (CE), necrotic, and total volumes; equivalent spherical CE width; several geometric measures of the CE ‘rim’ geometry and a set of image texture measures. The significance of the results was studied using Kaplan-Meier and Cox proportional hazards analysis. Correlations were assessed using Spearman correlation coefficients.

**Results:**

Kaplan-Meier and Cox proportional hazards analysis showed that total, CE and inner volume (p = 0.019, HR = 4.258) and geometric heterogeneity of the CE areas (p = 0.011, HR = 3.931) were significant parameters identifying response to BVZ. The group of patients with either regular CE areas (small geometric heterogeneity, median difference survival 15.88 months, p = 0.011) or those with small necrotic volume (median survival difference 14.50 months, p = 0.047) benefited substantially from BVZ.

**Conclusion:**

Imaging biomarkers related to the irregularity of contrast enhancing areas and the necrotic volume were able to discriminate GBM patients with a substantial survival benefit from BVZ. A prospective study is needed to validate our results.

## Introduction

Glioblastoma (GBM) is the most frequent type of malignant primary brain tumor and the most lethal type. The standard of care includes maximal safe surgery plus concurrent treatment with temozolomide (TMZ) and radiotherapy followed by maintenance TMZ, what leads to a median survival of 14.6 months [1].

Since GBM is one of the most vascularized human tumors it was thought that antiangiogenic treatment approaches might offer an alternative or complementary treatment strategy. Specifically, bevacizumab (BVZ), a humanized monoclonal antibody, has been in the spotlight of antiangiogenic approaches for several years [2].

Three randomized comparative phase III trials, two for newly diagnosed patients (AVAGLIO {ClinicalTrials.gov number NCT00943826} and RTOG08025 {ClinicalTrials.gov number NCT00884741}) [3,4] and one for recurrent GBMs (EORTC 26101{ClinicalTrials.gov number NCT01290939}) [5], reported negative results for the primary endpoint of overall survival (OS) but significant positive results for the BVZ arm in terms of progression-free survival (PFS) and overall response rate (ORR).

Nevertheless, there is a widespread, albeit statistically unsupported, perception that some patients do benefit from BVZ and that a response to BVZ predicts longer survival [6]. In fact, a population-based report highlighted an increase in survival among US GBM patients only attributable to the generalized use of BVZ in the recurrent setting following FDA approval [7].

Unfortunately, there is no reliable way to preselect patients who are most likely to respond to BVZ. Several studies have examined the possibility of selecting patients based on their molecular profile [8, 9] or on an early detection of response by image analysis [10,11,12,13].

Preoperative Magnetic resonance imaging (MRI) is routinely used for diagnosis, treatment planning, response evaluation and follow-up. Typical GBM appearance upon diagnosis on MRI consists of an enhancing ring mass with central non-enhancing core of necrosis observed mainly on contrast enhanced T1-weighted images; this is surrounded by an area of signal hyperintensity on fluid-attenuated inversion recovery FLAIR or T2 images representing edema that is well known to contain infiltrated tumor cells.

The areas of signal hyperintensity, were the contrast agent is released, indicate abnormal vessel permeability due to either the breakup of the blood-brain-barrier or the presence of inmature tumoral vessels. Typically, those areas have enhanced perfusion and its presence related to angiogenic processes.

There is an increasing evidence supporting that the geometrical and textural properties of tumors can lead to novel imaging biomarkers of prognosis and response. Pérez-García et al [14] developed a mathematical model predicting a positive correlation between the tumor rim width as observed in the postcontrast T1-weighted MRIs and tumor’s aggressiveness. This was later validated on a set of 117 GBM patients [15]. Other works have studied classical geometrical (volumetric) measures obtained from pretreatment T1 images as prognostic “biomarkers” [16,17]. Also, textural features in brain cancer have been widely studied to investigate their prognostic value [18,19], discriminate tumor phenotypes [20] or automatically detection and classification of low and high grade glioma [21,22].

The applicability of this kind of analysis to assess response to antiangiogenic treatment has not been put forward before.

In this paper we characterized the geometry and texture of pretreatment contrast enhancing (CE) areas in T1 weighted MRI images and correlated the resulting measures with survival benefits from BVZ. Our goal was to find novel imaging biomarkers predictive of antiangiogenic treatment efficacy in GBM patients.

## Materials and Methods

### Patients

The GENOM009 trial {ClinicalTrials.gov number NCT01102595} was a multicenter, prospective trial of neo-adjuvant therapy including 93 unresected patients with diagnosed GBM [23]. Patients were randomized 1:1 to either the temozolomide (TMZ) or the BVZ (TMZ+BVZ) arm. In the TMZ arm, neoadjuvant treatment consisted of TMZ for 2 cycles and then concurrent treatment with radiation therapy plus TMZ. In the TMZ+BVZ arm, patients received the same regimens but with the addition of BVZ in the neoadjuvant stage and concurrent stage. In both arms, the concurrent stage was followed by a 28-day break in treatment and then by adjuvant treatment with TMZ for six cycles without BVZ. The primary endpoint was investigator-assessed response after the neoadjuvant stage (week 9) according to the Response Assessment in Neuro-Oncology (RANO) Criteria [24]. Secondary endpoints were PFS, OS, 1-year survival among others. Results of this trial have been recently published showing that the addition of BVZ conferred a higher response rate (22.9% vs 6.7%; P<0.001) and greater clinical benefit (60.4% vs 24.5%; P<0.001). These results were confirmed upon centralized blinded review. PFS (4.8 vs 2.2 months) and OS (10.6 vs 7.7 months) were also longer in patients treated with the combination of TMZ+BVZ, though the difference did not reach statistical significance.

The inclusion criteria for this work were: availability of all of the relevant clinical variables (age, overall survival, etc.) and availability of high quality (no movement or other artifacts) pretreatment postcontrast T1-weighted MRIs. Multifocal GBMs and tumors without contrast enhancement were excluded from the study. Overall survival was measured from time of surgery to patient’s last contact or death.

High-resolution pre-surgery postcontrast T1-weighted MRIs were available for 53 patients, of which 13 were discarded due to either no contrast enhancement, diffuse data or presence of MRI noise. Therefore, 40 patients complied with inclusion criteria, 17 in the TMZ+BVZ arm and 23 in the TMZ arm. Patients' characteristics are summarized in Table 1.

**Table 1:** Summary of patient characteristics, MRI data and volumetric parameters for the 40 patients included in the study.

***Patient characteristics***	
Age (median, range)	64 (36–74)
Sex	21 Male (52.50%)
	19 Female (47.50%)
Survival (median, range)	10.78 months (2.30–52.33)
Treatment group	17 Temozolomide+Bevacizumab (42.50%)
	23 Temozolomide (57.50%)
***MRI characteristics (mean and range)***	
Pixel spacing (mm)	0.67 (0.43–1.04)
Slice thickness (mm)	2.54 (0.80–6.00)
Slices per patient	142 (16–301)
***Relevant volumetric parameters (mean and range)***	
Tumor volume (*V*) (cm^3^)	28.32 (0.99–79.23)
Contrast enhancing volume (*V*_CE_) (cm^3^)	19.99 (0.99–68.11)
Maximal tumor diameter (cm)	5.55 (1.48–10.57)
Spherical rim width (cm)	0.67 (0.25–2.07)

Table 2 lists patients age [25], Performance Status, Barthel index [26], and macroscopic tumor volume [15] for the different subgroups used in this study. There a high similarity between the different groups in terms of classical prognostic variables.

**Table 2:** Comparison of classical prognostic biomarkers values between the different patient subgroups.

	Genom009	Present study	BVZ+ (present study)	BVZ- (present study)
**Number of patients**	93	40	17	23
**Median age (Interquartile range) [Interval]**	63 (13.00)[36, 79]	64 (14.50)[36, 74]	63 (8.00)[51, 74]	64 (15.00)[36, 74]
**Average Performance Status(Standard deviation)[Interval]**	1.09(0.69)[0.0, 2.0]	1.00(0.78)[0.0, 2.0]	0.82(0.81)[0.0, 2.0]	1.13(0.76)[0.0, 2.0]
**Average Barthel index(Standard deviation) [Interval]**	86.89(15.26)[50.0, 100.0]	87.25(15.19)[55.0, 100.0]	90.00(15.10)[55.0, 100.0]	85.22(15.26)[55.0, 100.0]
**Average Tumor volume (Standard deviation) [Interval]**	-	28.32(23.31)[1.00, 79.23]	26.24(25.21)[1.00, 79.23]	29.85(22.26)[1.16, 69.18]

The GENOM-009 trial, including centralized review of imaging was approved by the institutional review boards (IRB) of each participating hospital and subsequently countersigned by the IRB of the Hospital Germans Trias i Pujol and approved by Spanish Agency for Medicines and Health Products (Agencia Española de Medicamentos y Productos Sanitarios, AEMPS). Written informed consent was obtained from all patients before registration. (EUDRACT: 2009-010337-45).

#### Image analysis

The DICOM files were imported into the scientific software package Matlab (R2015b, The MathWorks, Inc., Natick, MA, USA) and pre-processed using a semi-automatic image segmentation procedure. Segmented tumors were manually corrected by an image analysis expert with four years of experience with GBM MRIs.

#### Geometrical measures

After the segmentation, a set of geometrical 3D measures were computed automatically [15]. First, we calculated volumetric measures on the reconstructed tumors: the CE volume (*V*_*CE*_), the volume surrounded by the CE areas or inner volume (*V*_*I*_) and the total postcontrast T1 tumor volume (*V* = *V*_*CE*_ + *V*_*I*_). In most of the cases the inner volume corresponds to necrotic areas. Also the maximum tumor diameter in 3D *(d*_*max*_ 3D) and some measures of the tumor surface irregularity were computed.

The average size for the CE rim was called spherical rim width (*δ*_*s*_) because it can be obtained from a spherical approximation of the total and CE volume, using the formula

$$\delta_{s}=\sqrt[{3}]{\frac{3}{4\pi }}\left(\sqrt[{3}]{V}-\sqrt[{3}]{V_{I}}\right)\approx 0.62\left(\sqrt[{3}]{V}-\sqrt[{3}]{V_{I}}\right)$$

In order to characterize the structure of the CE rim we constructed the surfaces enclosing the CE areas (inner and outer). For every point in these surfaces the minimal distance to the other surface was computed, providing a set of distances, that was used to construct histograms of rim sizes. This histogram was characterized by many measures (median, mean, positions of the quartiles, etc). Of them, the most relevant found was the geometric heterogeneity of the CE rim width (*G*_*H*_). This is defined as the difference between the values of the quartile 4 and 3 of the distribution of the CE rim widths divided by the quartile 4, i.e.

$$G_{H}=\frac{\left(\delta_{4}-\delta_{3}\right)}{\delta_{4}}$$

The geometric heterogeneity uses the difference between the values of quartiles 4 and 3, i.e. the size of the region of the most asymmetric distances of the tumor, divided by the longest rim width in the tumor. This is a normalized measure of the tumor’s asymmetry that gets values in the range [0, 1].

**Fig 1:**
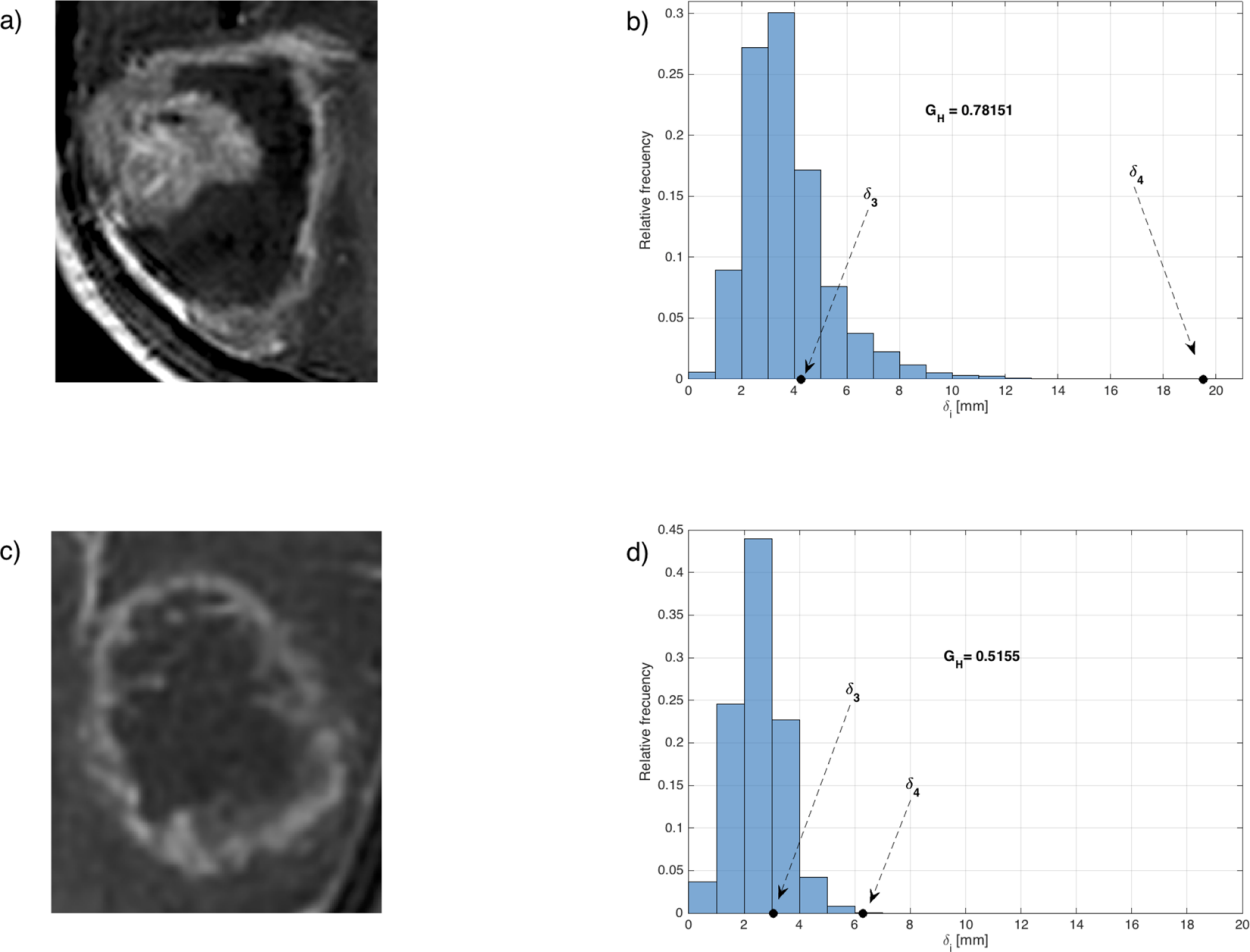
Visual examples of two tumors with different geometric heterogeneities. The first case corresponds to a rim-heterogeneous tumor (Fig 1A and 1B), having large geometric heterogeneity. The second case is a geometrically regular tumor (Fig 1C and 1D), with smaller geometric heterogeneity.

Fig 1 shows characteristic slices of two different tumors and the corresponding values of *G*_*H*_ obtained for each tumor. The first case (Fig 1A) corresponds to a heterogeneous tumor with a broad range of distances between the inner zones and the outer part of the CE rim. The second case (Fig 1C) corresponds to a geometrically more regular tumor. Fig 1B and 1D show their respective associated histograms of distances, from where the geometrical heterogeneity is computed. The presence of both large and small distances leads to more dispersed histograms, thus larger *G*_*H*_ (Fig 1B). More regular tumors lead to lower dispersion of widths and smaller values of *G*_*H*_ (Fig 1D).

#### Heterogeneity measures

A set of 16 3D heterogeneity textural measures was also computed automatically using Matlab software. The measures considered are based in the classical co-occurrence [27] and run-length matrices [28]. Co-occurrence matrix is a classical tool for heterogeneity computation providing information on *local* heterogeneity by describing the arrangements of pairs of elements (voxels) within the tumor. The run-length matrix characterizes groups of voxels within the tumor to provide information on *regional* heterogeneity. Table 3 lists the textural features considered.

**Table 3:** Definition of the heterogeneity measures computed in this study.

Type of measure	Name	Formula
Co-occurrence matrix	Entropy [27]	$-\sum_{i=1}^{N}\sum_{j=1}^{N}CM(i,j)\cdot \ln\left[CM(i,j)\right]$
Co-occurrence matrix	Homogeneity [27]	$\sum_{i=1}^{N}\sum_{j=1}^{N}\frac{CM(i,j)}{1+{\left(i-j\right)}^{2}}$
Co-occurrence matrix	Contrast [27]	$\sum_{i=1}^{N}\sum_{j=1}^{N}CM(i,j)\cdot {\left(i-j\right)}^{2}$
Co-occurrence matrix	Dissimilarity [27]	$\sum_{i=1}^{N}\sum_{j=1}^{N}CM(i,j)\cdot \left|i-j\right|$
Co-occurrence matrix	Uniformity [27]	$\sum_{i=1}^{N}\sum_{j=1}^{N}{\left[CM(i,j)\right]}^{2}$
Run-length matrix	Long Run Emphasis (LRE) [28]	$\frac{1}{n_{r}}\sum_{i=1}^{N}\sum_{j=1}^{M}RLM(i,j)\cdot j^{2}$
Run-length matrix	Short Run Emphasis (SRE) [28]	$\frac{1}{n_{r}}\sum_{i=1}^{N}\sum_{j=1}^{M}\frac{RLM(i,j)}{j^{2}}$
Run-length matrix	Low Gray-level Run Emphasis (LGRE) [28]	$\frac{1}{n_{r}}\sum_{i=1}^{N}\sum_{j=1}^{M}\frac{RLM(i,j)}{i^{2}}$
Run-length matrix	High Gray-level Run Emphasis (HGRE) [28]	$\frac{1}{n_{r}}\sum_{i=1}^{N}\sum_{j=1}^{M}RLM(i,j)\cdot i^{2}$
Run-length matrix	Short Run Low Gray-level Emphasis (SRLRE) [28]	$\frac{1}{n_{r}}\sum_{i=1}^{N}\sum_{j=1}^{M}\frac{RLM(i,j)}{i^{2}\cdot j^{2}}$
Run-length matrix	Short Run High Gray-level Emphasis (SRHGE) [28]	$\frac{1}{n_{r}}\sum_{i=1}^{N}\sum_{j=1}^{M}\frac{RLM(i,j)\cdot i^{2}}{j^{2}}$
Run-length matrix	Long Run Low Gray-level Emphasis (LRLGE) [28]	$\frac{1}{n_{r}}\sum_{i=1}^{N}\sum_{j=1}^{M}\frac{RLM(i,j)\cdot j^{2}}{i^{2}}$
Run-length matrix	Long Run High Gray-level Emphasis (LRHGE) [28]	$\frac{1}{n_{r}}\sum_{i=1}^{N}\sum_{j=1}^{M}RLM(i,j)\cdot i^{2}\cdot j^{2}$
Run-length matrix	Gray-Level Non-Uniformity (GLNU) [28]	$\frac{1}{n_{r}}\sum_{i=1}^{N}{\left(\sum_{j=1}^{M}RLM(i,j)\right)}^{2}$
Run-length matrix	Run-Length Non-Uniformity (RLNU) [28]	$\frac{1}{n_{r}}\sum_{j=1}^{M}{\left(\sum_{i=1}^{N}RLM(i,j)\right)}^{2}$
Run-length matrix	Run Percentage (RPC) [28]	$\frac{n_{r}}{\sum_{i=1}^{N}\sum_{j=1}^{M}RLM(i,j)\cdot j}$

For CM measures CM(i,j) stands for the co-occurrence matrix, N is the number of classes of gray-levels taken (32 in this study). For RLM measures RLM(i,j) is the run-length matrix, nr is the number of runs, N is the number of classes of gray-levels and M is the size in voxels of the largest region found.

#### Statistical methods

To identify geometric parameters associated with prognosis we used Kaplan–Meier plots and log-rank analysis. A 2-tailed significance level (p-value) of p<0.05 was applied. We tried to find measures separating patient populations in two groups with significant differences in terms of survival. To do so we: (i) computed the p-values for the full range of thresholds of each variable considered in the study, (ii) accepted only thresholds that separated the populations on reasonably-sized subgroups, and (iii) looked minimum p-values located in low p-value regions of the parameter space (in order to discard purely statistical fluctuations). For each population splitting, we computed the hazard ratio (HR) as an indicator of risk by using a single-variable Cox proportional hazards regression analysis.

In order to identify measures with similar information, we computed the correlations between the significant variables of the previous analysis. Because of the normality analysis, Spearman's correlation coefficient was considered to study the relation between independent quantitative variables. Correlation coefficient values <0.1 were taking as indicators of no correlation between the variables while values >0.7 were taken as indicators of strong correlation between the variables.

## Results

We segmented and geometrically analyzed brain imaging datasets belonging to the 40 patients included in our study (Table 1). By means of Kaplan-Meier analysis, we first compared survival of patients receiving TMZ+BVZ with those only receiving TMZ. While there seems to be a better survival for patients in the TMZ+BVZ arm, the result was statistically non-significant (p = 0.107) for the subset of patients from the Genom009 trial included in this study, what is in line with the clinical trial results.

Next, we studied the geometrical and heterogeneity measures described in the methods section using Kaplan-Meier curves and Cox proportional hazard analysis. Patients were split into subgroups as described in the methods section. Table 4 summarizes the results for the different population splits using the mean, the median and the optimal thresholds used for each significant variable. Thresholds were computed for the BVZ group and then the statistical analysis was performed for both arms.

**Table 4:** Summary of univariate Cox and Kaplan-Meier analysis for the more representative variables included in the study. Significant results are boldfaced.

	17 patients (BVZ)		23 patients (No BVZ)	
Variables	HR, (CI-95%)	p-value	HR, (CI-95%)	p-value
**Age (years)**				
Age (< = 65.00 vs >65.00) (years)	2.721 (0.879, 8.425)	0.072	2.331 (0.864, 6.285)	0.084
Mean: Age (< = 63.00 vs >63.00) (years)	2.485 (0.846, 7.299)	0.088	1.341 (0.573, 3.138)	0.496
Median: Age (< = 63.00 vs >63.00) (years)	2.485 (0.846, 7.299)	0.088	1.341 (0.573, 3.138)	0.496
***V*_*I*_ (cm^3^) (Necrotic volume)**				
*V*_*I*_ (< = 2.50 vs >2.50) (cm^3^)	**4.258 (1.160. 15.635)**	**0.019**	2.140 (0.855, 5.361)	0.096
Mean: *V*_*I*_ (< = 7.32 vs >7.32) (cm^3^)	1.099 (0.389, 3.106)	0.858	1.843 (0.757, 4.488)	0.171
Median: *V*_*I*_ (< = 4.49 vs >4.49) (cm^3^)	1.549 (0.552, 4.348)	0.402	1.843 (0.757, 4.488)	0.171
***V* (cm^3^) (Total volume)**				
*V* (< = 5.00 vs >5.00) (cm^3^)	**4.258 (1.160. 15.635)**	**0.019**	1.502 (0.432, 5.228)	0.519
Mean: *V* (< = 26.24 vs >26.24) (cm^3^)	1.927 (0.635, 5.850)	0.239	1.453 (0.615, 3.435)	0.391
Median: *V* (< = 21.28 vs >21.28)(cm^3^)	2.676 (0.845, 8.471)	0.083	1.824 (0.749, 4.443)	0.178
***V*_*CE*_ (cm^3^) (CE volume)**				
*V*_*CE*_ (< = 3.50 vs >3.50) (cm^3^)	**4.258 (1.160. 15.635)**	**0.019**	1.502 (0.432, 5.228)	0.519
Mean: *V*_*CE*_ (< = 18.91 vs >18.91) (cm^3^)	2.676 (0.845, 8.471)	0.083	1.288 (0.545, 3.047)	0.562
Median: *V*_*CE*_ (< = 14.40 vs >14.01) (cm^3^)	**3.445 (1.008, 11.770)**	**0.038**	1.824 (0.749, 4.443)	0.178
**G_H_ (Geometric heterogeneity)**				
*G*_*H*_ (< = 0.605 vs >0.605)	**3.931 (1.289, 11.985)**	**0.011**	1.070 (0.441, 2.596)	0.881
Mean: *G*_*H*_ (< = 0.608 vs >0.608)	**3.050 (1.050. 8.856)**	**0.032**	1.070 (0.441, 2.596)	0.881
Median: *G*_*H*_ (< = 0.623 vs >0.623)	**3.050 (1.050. 8.856)**	**0.032**	1.268 (0.362, 4.438)	0.709

The best thresholds for *V*, *V*_*I*_, *V*_*CE*_ and *G*_*H*_ were found to be 5 cm^3^ (p = 0.019), 2.5 cm^3^ (p = 0.019), 3.5 cm^3^ (p = 0.019) and 0.605 (p = 0.011) respectively (see Table 4). The increases in the median survival times for the favorable subgroups were of 19.72 months (*V*<5 cm^3^, *V*_*I*_<2.5 cm^3^ and *V*_*CE*_<3.5 cm^3^) and 19.40 months (*G*_*H*_<0.605). None of the studied image heterogeneity features reached statistical significance.

**Fig 2:**
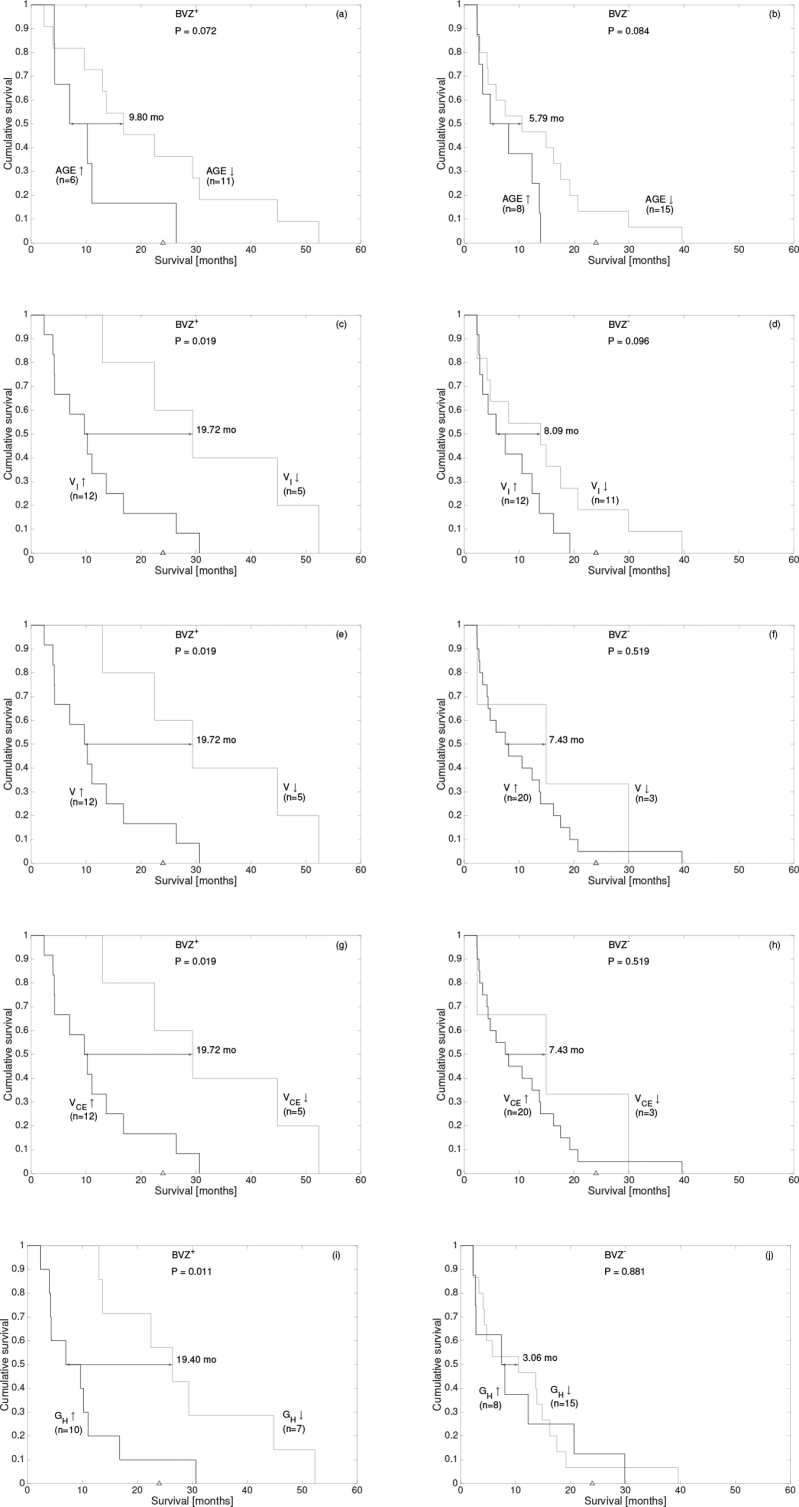
Kaplan-Meier curves for the each significant geometrical variable (plus age, that was marginally significant) using the thresholds of Table 4: (a,b) age, (c,d) *V*_*I*_, (e,f) *V*, (g,h) *V*_*CE*_ and (i,j) *G*_*H*_. The median differences, p-values and number of patients per group are indicated in each subplot. Panels (a, c, e, g and i) correspond to patients in the BVZ+TMZ arm while panels (b, d, f, h and j) correspond to the TMZ arm.

Fig 2C, 2E, 2G and 2I show Kaplan-Meier survival curves for the significant variables of our study. The three different volumetric measures considered (*V*, *V*_*I*_ and *V*_*CE*_) led to the same population splitting and thus the same Kaplan-Meier curves (shown in Fig 2C, 2E and 2G). This is due to the limited number of patients in the study and high correlation between the volumetric features. We also show the best threshold for the age (Fig 2A) although this variable was only marginally significant in our study. In order to discard purely statistical fluctuations, we studied the same computed thresholds on the TMZ only arm. Fig 2B, 2D, 2F, 2H and 2J show the corresponding Kaplan-Meier survival curves.

**Fig 3:**
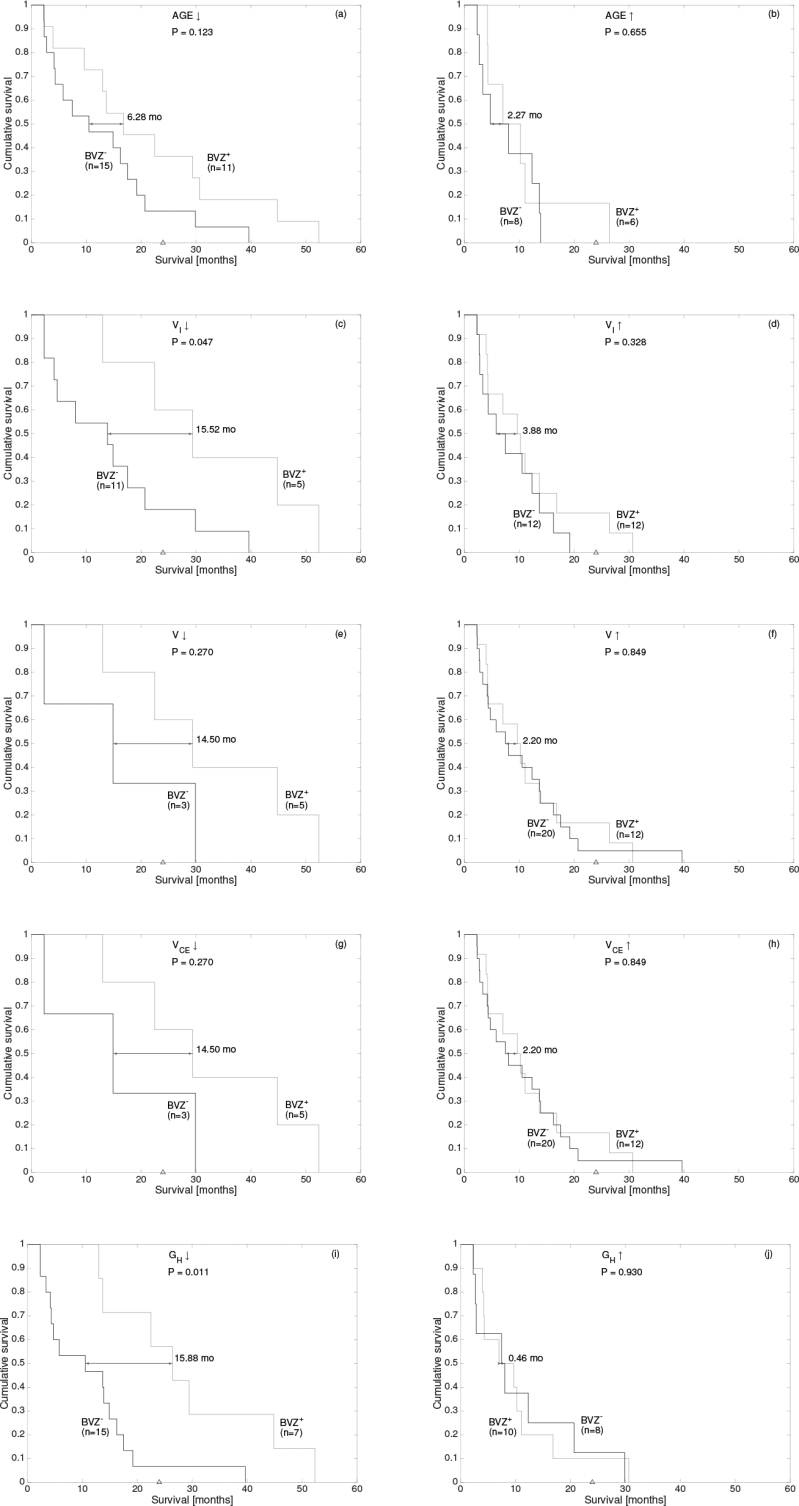
Kaplan-Meier curves comparing survival of subgroups of patients below or above the optimal thresholds shown in Table 4: (a,b) age, (c,d) *V*_*I*_, (e,f) *V*, (g,h) *V*_*CE*_ and (i,j) *G*_*H*_. The median differences, p-values and number of patients in each subgroup are indicated.

The next step in our analysis was to compare patients having the same geometrical characteristics and study whether BVZ provides any significant benefits. To do so, for each variable found to be significant in the previous analysis, we separated the initial 40 patients between those being above or below the selected best threshold. Then, we used Kaplan-Meier analysis to compare patients in both trial arms. Fig 3 summarizes our results.

The outstanding feature of this analysis was the contrast enhancing rim geometric heterogeneity *G*_*H*_ (Fig 3I and 3J). Patients having a small *G*_*H*_ had a significant strong benefit from BVZ (*p* = 0.011, Fig 3I) with a median survival difference of 15.88 months. On the other hand, patients with large *G*_*H*_ (*p* = 0.930. Fig 3J) did not present any benefit from BVZ.

The necrotic volume *V*_*I*_ also showed significant results in terms of survival (Fig 3C and 3D). Patients having a small *V*_*I*_ strongly benefited from BVZ (*p* = 0.047, Fig 3C) with a median survival of 15.52 months. On the other hand, patients with large *V*_*I*_ (*p* = 0.328. Fig 3D) did not present any benefit from BVZ.

As next step, we computed the Spearman’s correlation coefficient and the associated p between all significant variables (Table 5). It is relevant to point out that the volumetric variables (*V*_*CE*_, *V*_*I*_, *V*) were mutually correlated and weakly (but significantly in most cases) correlated with the geometric heterogeneity *G*_*H*_.

**Table 5:** Spearman correlation coefficients between every pair of significant variables in our study. Boldfaced numbers indicate significant correlations (p<0.05).

	*V*_*I*_	*V*	*V*_*CE*_	G_H_
***V_I_***	**1**	**0.790**	**0.670**	**0.522**
***V***		**1**	**0.963**	**0.322**
***V_CE_***			**1**	**0.253**
**G**_**H**_				**1**

Table 6 shows the computed values of the geometric heterogeneity and the necrotic volumes for all patients included in this study, together with their overall survival and O(6)-Methylguanine-DNA-methyltransferase (MGMT) status. Patients in the first part of the table (those taking only TMZ) having large overall survival could be mostly identified by the presence of the MGMT methilation, due to its relation with the response to temozolomide. On the other hand, patients in the BVZ group (second part of Table 6) with long survivals were strongly identified by *G*_*H*_.

**Table 6:** Overall survival, *G*_*H*_, *V*_*I*_ and MGMT status (when available) for all of the patients included in the study. Within each group (BVZ+ or BVZ-) patients are ordered by survival. Grey cells indicate patients in the favourable groups: either small *G*_*H*_ (dark grey) or unmethilated MGMT (light grey).

	BVZ-						BVZ+		
Patient	OS	*V*_*I*_	*G*_*H*_	MGMT	Patient	OS	*V*_*I*_	*G*_*H*_	MGMT
1	70	14.182	0.611	1	24	72	2.979	0.752	1
2	71	0.070	0.444	-	25	121	4.135	0.623	0
3	71	0.439	0.292	0	26	128	3.866	0.635	1
4	82	39.114	0.719	0	27	131	12.598	0.607	0
5	86	8.699	0.609	0	28	213	6.401	0.792	1
6	102	3.640	0.562	0	29	294	6.875	0.633	1
7	126	1.422	0.395	0	30	311	30.054	0.721	-
8	132	8.243	0.572	0	31	336	19.244	0.698	0
9	144	2.384	0.467	0	32	395	0.220	0.588	0
10	176	10.236	0.589	0	33	416	4.486	0.544	1
11	227	16.513	0.621	-	34	511	11.819	0.662	-
12	245	2.349	0.646	-	35	683	0.011	0.368	-
13	320	14.882	0.505	-	36	803	11.677	0.531	1
14	375	30.470	0.623	0	37	894	1.884	0.601	1
15	416	3.695	0.453	1	38	932	7.473	0.723	-
16	422	0.872	0.539	1	39	1363	0.093	0.346	1
17	453	0.054	0.235	0	40	1592	0.682	0.509	1
18	494	26.232	0.589	1					
19	533	1.527	0.412	1					
20	584	20.928	0.581	-					
21	629	0.825	0.610	-					
22	909	0.353	0.725	-					
23	1205	1.546	0.402	0					

## Discussion

Personalized medicine is becoming one of the major research topics in medicine and specifically in oncology [29]. Researchers have discovered genes that harbor variations contributing to human cancers, identified genetic variability in patients’ responses to different treatments, and begun to target the causes of some tumors. This requires the use of diagnostic tests able to predict patients’ responses to the therapies. Imaging biomarkers in different types of cancers [30,31] and specifically in GBM [32,33] are receiving a lot of attention recently as an alternative or complement to the more ‘standard’ tests based on genetics or other molecular mechanisms.

Experimental data indicate that the most important angiogenic signaling pathway in gliomas involves the vascular endothelial growth factor (VEGF) and its receptor VEGFR-2. BVZ is a human recombinant monoclonal antibody specific for the VEGF ligand that is being used to treat recurrent GBM in spite of the scarcity of direct evidence of increased survival with such treatment compared to other existing alternatives [34].

Imaging biomarkers have been found in GBM to determine prognosis [15,19,32,33,35] and to assess the response to antiangiogenic therapies [36]. Specifically, to distinguish progression from pseudoprogression or to provide early indications of treatment response in recurrent GBMs [12,13,31].

Our goal in this study was to test if a set of geometrical [15] and heterogeneity [19,27,28] features obtained from pretreatment postcontrast T1 weighted MRIs, some of them found previously to have a prognostic value, were predictors of response to antiangiogenic therapies.

When the group of patients receiving BVZ was considered, and for several geometrical variables, we found thresholds splitting this group into subgroups with significant differences in survival. When the same threshold was found to split the population of patients receiving only TMZ there were no significant differences between both subgroups. This means that the thresholds found are not just indicating a better prognosis group for the whole GBM patient population but instead reflecting a different response to the therapy in the different groups.

The analysis developed points out to two geometric parameters: the necrotic volume *V*_*I*_ and the geometric heterogeneity *G*_*H*_ as potential predictors of BVZ effectivity. Due to the limited number of patients available *V*_*I*_ was only marginally significant what points out the need for further prospective studies.

It is remarkable that patients with small values of the geometric heterogeneity of the contrast enhancing rim, in many cases being the same ones as those with small necrotic volumes, were the ones having more benefits from therapy. These tumors (small *G*_*H*_ and *V*_*I*_) display uniform contrast enhancement and small necrotic cores with rounded shapes. It has been demonstrated using mathematical models that that harsh tumor microenvironment conditions exert a dramatic selective force on the tumor, which grows as an invasive mass with fingering margins, dominated by a few clones with aggressive traits [37]. In the specific case of GBM those conditions arise after hypoxic events, that lead to the generation of necrotic areas and the acceleration of tumor growth and the development of more aggressive and infiltrative phenotypes [38,39]. Thus, in line with recent mathematical models [14], we think that these tumors are probably in the early steps of their natural history and are still more dependent on angiogenesis for their development.

In contrast, mild microenvironment conditions allow clones with similar aggressive traits to coexist with less aggressive phenotypes in a heterogeneous tumor mass with smooth, noninvasive margins [37]. Thus, the genetic make-up of a cancer cell may realize its invasive potential through a clonal evolution process driven by definable microenvironmental selective forces. The presence of necrosis and/or large values of the geometric heterogeneity would be a macroscopic reflection of these microscopic processes.

Indeed, the mesenchimal GBM subtype is known to generate large necrotic areas, have higher infiltration and could be less dependent on angiogenesis than tumors with the proliferative molecular subtype [40]. For our patients genomic data was not available but it would be very interesting to correlate these macroscopic features with genomic data on the light of the GBM molecular classification.

As mentioned before, the main limitation of our study is the small number of patients, due to the restrictive inclusion criteria imposed. Specifically, high resolution images (3D T1) are necessary to obtain reliable values for the geometrical parameters in general and very specifically to compute *G*_*H*_. This is why Genom009 was a good candidate trial to include its patients in our study, since neoadjuvant treatment (BEV and TMZ or TMZ) was administered as first-line treatment before radiotherapy, thus, removing the effects of radiotherapy over the blood brain barrier that produce secondary changes in contrast enhancement and difficult the response assessment as the study was performed over basal, pretreatment MRI. A prospective study design with high resolution images, in agreement with current recommendations [41], is necessary to confirm the potential clinical applicability of the imaging biomarkers defined here.

## Conclusions

Two geometrical features obtained from high resolution postcontrast and pretreatment MRI T1-weighted images were predictive of benefit from BVZ: the geometric heterogeneity of the contrast-enhancing rim and the necrotic volume. Our results suggest that this drug may have a substantial potential benefit on a subset of patients with tumors having regular geometries or very small necrotic volumes. The measurement, study and validation of these characteristics may be key to plan treatment and predict response and evolution of GBM patients. A prospective study is necessary to corroborate our results.

## References

[1] Stupp R, Mason WP, van den Bent MJ, Weller M, Ficher B, Taphoorn MJB, et al Radiotherapy plus Concomitant and Adjuvant Temozolomide for Glioblastoma, N Engl J Med. 2005;352:987–96. 15758009 10.1056/NEJMoa043330

[2] Weathers SP, de Groot J. VEGF manipulation in glioblastoma, Oncology. 2015;29:720–27. 26470893

[3] Chinot OL, Wick W, Mason W, Henriksson R, Saran F, Nishikawa R, et al Bevacizumab plus radiotherapy-temozolomide for newly diagnosed glioblastoma. N Engl J Med. 2014;370:709–22. 10.1056/NEJMoa1308345 24552318

[4] Gilbert MR, Dignam JJ, Armstrong TS, Wefel JS, Blumenthal DT, Vogelbaum MA, et al A randomized trial of bevacizumab for newly diagnosed glioblastoma. N Engl J Med. 2014;370:699–708. 10.1056/NEJMoa1308573 24552317 PMC4201043

[5] Wick W, Brander A, Gorlia T, Bendszus M, Sahm F, Taal W, et al Phase III trial exploring the combination of bevacizumab and lomustine in patients with first recurrence of a glioblastoma: the EORTC 26101 trial. Neuro Oncol. 2015;17 Suppl 5:v1–v1.

[6] Prados M, Cloughesy T, Samant M, Fang L, Wen PY, Mikkelsen T, et al Response as a predictor of survival in patients with recurrent glioblastoma treated with bevacizumab. Neuro Oncol. 2011;13:143–51. 10.1093/neuonc/noq151 21084434 PMC3018914

[7] Johnson DR, Leeper HE, Uhm JH. Glioblastoma survival in the United States improved after Food and Drug Administration approval of bevacizumab: a population-based analysis. Cancer. 2013;119:3489–95. 10.1002/cncr.28259 23868553

[8] Sandmann T, Bourgon R, Garcia J, Li C, Cloughesy T, Chinot OL, et al Patients with proneural glioblastoma may derive overall survival benefit from the addition of bevacizumab to first-line radiotherapy and temozolomide: retrospective analysis of the AVAglio Trial. J Clin Oncol. 2015;33:2735–44. 10.1200/JCO.2015.61.5005 26124478 PMC5015426

[9] Erdem-Eraslan L, van den Bent MJ, Hoogstrate Y, Naz-Khan H, Stubbs A, van der Spek P, et al Identification of patients with recurrent glioblastoma who may benefit from combined bevacizumab and CCNU therapy: a report from the BELOB trial. Cancer Res. 2016;76:525–34. 10.1158/0008-5472.CAN-15-0776 26762204

[10] Pope WB, Kim HJ, Huo J, Alger J, Brown MS, Gjertson D, et al Recurrent glioblastoma multiforme: ADC histogram analysis predicts response to bevacizumab treatment. Radiology. 2009;252:182–9. 10.1148/radiol.2521081534 19561256

[11] Lu-Emerson C, Duda DG, Emblem KE, Taylor JW, Gerstner ER, Loeffler JS, et al Lessons from anti-vascular endothelial growth factor and anti-vascular endothelial growth factor receptor trials in patients with Glioblastoma. J Clin Oncol. 2015;33:1197–213. 10.1200/JCO.2014.55.9575 25713439 PMC4517055

[12] Aquino D, Di Stefano AL, Scotti A, Cuppini L, Anghileri E, Finocchiaro G, et al Parametric response maps of perfusion MRI may identify recurrent glioblastomas responsive to bevacizumab and irinotecan. PLoS ONE. 2014;9:e90535 10.1371/journal.pone.0090535 24675671 PMC3968002

[13] Pope WB. Evidence for rCBV as an early response marker following bevacizumab treatment. Neuro Oncol. 2015;17:1539–40. 10.1093/neuonc/nov199 26453577 PMC4648311

[14] Pérez-García VM, Calvo GF, Belmonte-Beitia J, Diego D, Pérez-Romasanta LA. Bright solitary waves in malignant gliomas. Phys Rev E. 2011;84:021921. 10.1103/PhysRevE.84.021921 21929033

[15] Pérez-Beteta J, Martínez-González A, Molina D, Amo-Salas M, Luque B, Arregui E et al Glioblastoma: Does the pretreatment geometry matter? A postcontrast T1 MRI-based study. Eur Radiol. 2016; 10.1007/s00330-016-4453-9 27329522

[16] Mazurowski MA, Zhang J, Peters KB, Hobbs H. Computer extracted MR imaging features are associated with survival in glioblastoma patients. J Neurooncol. 2014;120:483–8. 10.1007/s11060-014-1580-5 25151504

[17] Zinn PO, Sathyan P, Mahajan B, Bruyere J, Hegi M, Majumder S et al A novel volume-age-KPS (VAK) glioblastoma classification identifies a prognostic cognate microRNA-gene signature. PLoS One. 2012;7:e41522 10.1371/journal.pone.0041522 22870228 PMC3411674

[18] Upadhaya T, Morvan Y, Stindel E, Le Reste PJ, Hatt M. Prognostic value of multimodal MRI tumour features in Glioblastoma multiforme using textural features analysis. Biomedical Imaging IEEE 12th International Symposium. 2015; 50–4.

[19] Molina D, Pérez-Beteta J, Luque B, Arregui E, Calvo M, Borrás JM, et al Tumour heterogeneity in glioblastoma assessed by MRI texture analysis: a potential marker of survival. British Journal of Radiology 2016; 86, 20160242 10.1259/bjr.20160242 PMC5124892 27319577

[20] Chaddad A, Zinn PO, Colen RR. Quantitative Texture Analysis for Glioblastoma Phenotypes Discrimination. International Conference on Control, Decision and Information Technologies (CoDIT). 2014; 605–8.

[21] Ryu YJ, Choi SH, Park SJ, Yun TJ, Kim J-H, Sohn C-H. Glioma: Application of Whole-Tumour Texture Analysis of Diffusion-Weighted Imaging for the Evaluation of Tumour Heterogeneity. PLoS ONE. 2014; 9: e108335 10.1371/journal.pone.0108335 25268588 PMC4182447

[22] Resmi A, Tessamma T. Automatic Detection and Classification of Glioma Tumours using Statistical Features. Int Jl Emerging Technol. 2014; 7:8–14.

[23] Balana C, De Las Penas R, Sepulveda JM, Gil-Gil MJ, Luque R, Gallego O, et al Bevacizumab and temozolomide versus temozolomide alone as neoadjuvant treatment in unresected glioblastoma: the GENOM009 randomized phase II trial. J Neuro Oncol. 2016;127:569–79. 10.1007/s11060-016-2065-5 26847813

[24] Wen PY, Macdonald DR, Reardon DA, Cloughesy TF, Sorensen AG, Galanis E et al Updated response assessment criteria for high-grade gliomas: response assessment in neuro-oncology working group. J Clin Oncol. 2010;28:1963–72. 10.1200/JCO.2009.26.3541 20231676

[25] Lamborn KR, Chang SM, Prados MD. Prognostic factors for survival of patients with glioblastoma: Recursive partitioning analysis. Neuro-Oncology. 2004;6:227–235. 15279715 10.1215/S1152851703000620 PMC1871999

[26] Flechl B, Ackerl M, Sax C, Dieckman K, Crevenna R, Gaiger A et al Neurocognitive and sociodemographic functioning of glioblastoma long-term survivors. J Neurooncol. 2012; 109:331–339. 10.1007/s11060-012-0897-1 22644537

[27] Tixier F, Hatt M, Le Rest CC, Le Pogam A, Corcos L, Visvikis D. Reproducibility of tumour putake heterogeneity characterization through textural feature analysis in 18f-fdg pet imaging. J Nucl Med. 2012; 53(5):693–700. 10.2967/jnumed.111.099127 22454484 PMC3779464

[28] Tixier F, Le Rest CC, Hat M, Algarghach N, Pradier O, Metges JP et al Intratumour heterogeneity characterized by textural features on baseline 18F-FDT PET images predicts response to concomitant radiochemotherapy in esophageal cancer. J Nucl Med. 2011; 52(3):369–78. 10.2967/jnumed.110.082404 21321270 PMC3789272

[29] Hamburg MA, Collins FS. The path to personalized medicine. N Engl J Med. 2010;363:301–4. 10.1056/NEJMp1006304 20551152

[30] Mankoff DA, Pryma DA, Clark AS. Molecular imaging biomarkers for oncology clinical trials. J Nucl Med. 2014;55:1–4. 24614222 10.2967/jnumed.113.126128

[31] Zinn PO, Mahmood Z, Elbanan MG, Colen RR. Imaging Genomics in Gliomas. Cancer J. 2015;21:225–34. 10.1097/PPO.0000000000000120 26049703

[32] Johnson DR O'Neill BP. Glioblastoma survival in the United States before and during the temozolomide era. J Neurooncol. 2012;107:359–64. 10.1007/s11060-011-0749-4 22045118

[33] Hutterer M, Hattingen E, Palm C, Proescholdt MA, Hau P. Current standards and new concepts in MRI and PET response assessment of antiangiogenic therapies in high-grade glioma patients. Neur Oncol. 2015;17:784–800. 10.1093/neuonc/nou322 PMC4483117 25543124

[34] Ellingson BM. Radiogenomics and imaging phenotypes in glioblastoma: novel observations and correlation with molecular characteristics. Curr Neurol Neurosci Rep. 2015;15:506 10.1007/s11910-014-0506-0 25410316

[35] Hygino da Cruz LC, Rodriguez I, Domingues RC, Gasparetto EL, Sorensen AG. Pseudoprogression and pseudoresponse: imaging challenges in the assessment of posttreatment glioma. Am J Neuro Radiol. 2011;32:1978–85. 10.3174/ajnr.A2397 PMC7964401 21393407

[36] O'Connor J, Jackson A, Asselin MC, Buckley DL, Parker GJ, Jayson GC. Quantitative imaging biomarkers in the clinical development of targeted therapeutics: current and future perspectives. Lancet Oncol. 2008;9:766–76. 10.1016/S1470-2045(08)70196-7 18672212

[37] Anderson AR, Weaver A, Cummings PT, Quaranta V, Tumor Morphology and Phenotypic Evolution Driven by Selective Pressure from the Microenvironment, Cell 2006; 127: 905–915. 17129778 10.1016/j.cell.2006.09.042

[38] Martínez-González A, Calvo GF, Pérez Romasanta LA, Pérez-García VM. Hypoxic Cell Waves Around Necrotic Cores in Glioblastoma: A Biomathematical Model and Its Therapeutic Implications. Bulletin of Mathematical Biology. 2012; 74:2875–2896. 10.1007/s11538-012-9786-1 23151957 PMC3510407

[39] Pardo R, Martínez-González A, Pérez-García VM. Nonlinear ghost waves accelerate the progression of high-grade brain tumors, Commun Nonlin Sci Numer Simul. 2016; 39: 360–380.

[40] Verhaak RGW, Hoadley KA, Purdom E, Wang V, Qi Y, Wilkerson MD, et al An integrated genomic analysis identifies clinically relevant subtypes of glioblastoma characterized by abnormalities in PDGFRA, IDH1, EGFR and NF1. Cancer Cell. 2010;17:157–73. 10.1016/j.ccr.2009.12.020 PMC2818769 20129251

[41] Ellingson BJ, Bendszus M, Boxerman J, Barboriak D, Erickson BJ, Smits M, et al Consensus recommendations for a standardized Brain Tumor Imaging Protocol in Clinical Trials. Neur Oncol. 2015;17:1188–98. 10.1093/neuonc/nov095 PMC4588759 26250565

